# The Confounders of Cancer Immunotherapy: Roles of Lifestyle, Metabolic Disorders and Sociological Factors

**DOI:** 10.3390/cancers12102983

**Published:** 2020-10-15

**Authors:** Ravindra Pramod Deshpande, Sambad Sharma, Kounosuke Watabe

**Affiliations:** Department of Cancer Biology, Wake Forest Baptist Medical Center, Winston-Salem, NC 27157, USA; rdeshpan@wakehealth.edu (R.P.D.); ssharma@wakehealth.edu (S.S.)

**Keywords:** immunotherapy, anti PD-L1, anti-CTLA, diet, obesity, diabetes, circadian rhythms

## Abstract

**Simple Summary:**

The human immune system is robustly equipped to keep unnatural cell growth in check in the body to suppress cancer progression. However, the cells and molecules of the immune system responsible for preventing cancer growth are often severely compromised in patients harboring this disease. Therefore, to elicit a functional immune response against cancer, scientists have developed antibodies, known as checkpoint inhibitors (CPI), which unleash the compromised immune cells and potentiate them with cancer killing ability. Although CPI revolutionized the treatment of certain cancers, many patients do not respond to the CPI and the treatment outcome varies disproportionately between cancer types. This review elaborates on how lifestyle, metabolic and sociological factors play a role in determining the outcome of CPI treatment. We also discuss potential ways to enhance CPI efficacy by mitigating the effect of these confounding variables.

**Abstract:**

Checkpoint blockade immunotherapy (CPI) is an effective treatment option for many types of cancers. Irrespective of its wide clinical implications, the overall efficacy remains unpredictable and even poor in certain pathologies such as breast cancer. Thus, it is imperative to understand the role of factors affecting its responsiveness. In this review, we provide an overview on the involvement of sociological factors, lifestyles and metabolic disorders in modulating the CPI response in patients from multiple malignancies. Lifestyle habits including exercise, and diet promoted therapeutic responsiveness while alcohol consumption mitigated the CPI effect by decreasing mutational burden and hampering antigen presentation by dendritic cells. Metabolic disorder such as obesity was recognized to enhance the PD-1 expression while diabetes and hypertension were consequences of CPI therapy rather than causes. Among the sociologic factors, sex and race positively influenced the CPI effectiveness on account of increased effector T cell activity and increased PD-1 expression while ageing impaired CPI responsiveness by decreasing functional T cell and increased toxicity. The combined effect of these factors was observed for obesity and gender, in which obese males had the most significant effect of CPI. Therefore these variables should be carefully considered before treating patients with CPI for optimal treatment outcome.

## 1. Introduction

T cells are the main effector cells for anti-tumor defense. T cell immune checkpoints are the regulators of T cell functionality and play a crucial role in maintaining peripheral tolerance and to prevent autoimmunity. T cell responsiveness is guided by the balance between co-stimulatory and co-inhibitory pathways. Immune checkpoints are co-employed by the tumor cells to escape the immune surveillance. Checkpoint blockade drugs work by unleashing the T cells to recognize and kill tumors. At present the most well studied checkpoint factors include Programmed cell death protein 1 (PD-1), cytotoxic T-lymphocyte-associated protein 4 (CTLA-4), Lymphocyte-activation gene 3 (LAG-3), T-cell immunoglobulin and mucin domain-3 (TIM-3) and T cell immunoreceptor with Ig and ITIM domains (TIGIT) [1,2].

Interactions between molecules expressed by immune- and tumor cells are involved during the mounting of the inhibitory response. Binding of PD-L1, expressed by cancer cells, to its cognate receptor PD-1 on T cells is one of such interaction known to deliver an inhibitory signal to T-cells leading to their dysfunction and exhaustion. Targeting the PD-1/PD-L1 checkpoint, one modality of checkpoint blockade immunotherapy (CPI), has been an established treatment for many cancers and has impacted the life expectancy and clinical outcome of many patients. Another CPI in clinical use targets the binding of CTLA4 to its ligands, expressed by antigen presenting cells. CTLA4 is expressed by the T cells and is a homologue of the co-stimulatory molecule, CD28. CD28 interacts with its ligands CD80 and CD86, expressed by antigen presenting cells and promotes costimulatory signaling. CTLA4 has a higher affinity for CD80 and CD86 than CD28, and its binding antagonizes the natural interaction to mediate the negative regulation of T cell activation function. In addition to CTLA, TIM3 and LAG3 are predominantly expressed by immune cells including CD8^+^, CD4^+^ and Treg cells and negatively regulate their proliferation, thus dampening the immunological response [3,4] (Table 1). Together, engagement of these inhibitory receptors leads to the downstream release of cytokines, hampering the neutralization of cancer cells by the immune system [5]. Therefore, CPI works to unleash the inhibition from functional immune cells to regain its anti-tumor activity.

In addition to T cells, other immune cells such as natural killer (NK) cells, macrophages and neutrophils also express checkpoint inhibitor proteins. NK cells are cytotoxic innate immune cells and they do not express antigen specific cell surface receptors [18]. Importantly, NK cells express LAG-3, TIM-3, PD-1 and TIGIT immunomodulatory receptors [19]. In glioblastoma, blockade of the TIGIT receptor, in combination with PD-1/PD-L1 inhibitors was shown to augment the anti-tumor effect of CPI treatment [20]. Another study demonstrated that interaction between tumor PD-L1 with PD-1 expressed by NK cells inhibits the antitumor responses of NK cells, leading to aggressive tumor growth [21]. Tumor associated macrophages (TAM) are the most abundant immune cells and are known to differentially regulate tumor progression [22,23]. TAM also express both PD-1 and PD-L1 on their cell surface [24]. The tumor supportive “M2” macrophages express higher levels of PD-1 as compared with tumor suppressive “M1” macrophages. The PD-1 positive macrophages were also shown to express less MHC-II and have compromised phagocytosis [25]. In a colon cancer model, it was demonstrated that in tumors treated with antibodies targeting PD-1 receptors, anti-PD-1 antibodies that are bound to T-cells were acquired by PD-1 negative TAMs. This acquisition of anti-PD-1 antibodies was dependent on the Fcγ-receptors (FcγRs) on macrophages that interacted with the Fc domain of the anti-PD1 antibodies. Blockade of FcγRs enhanced the effect of immunotherapy by prolonging the binding between PD-1 antibody and CD-8 T cells [26]. In addition, we have previously shown that M2 microglia, macrophages of the brain, upregulated the PD-L1 expression and promoted breast cancer brain metastasis by immune suppression [27]. Another immune cell that potentially effects the checkpoint therapy response is the neutrophil. Neutrophils present in the tumor microenvironment are known to express PD-L1 and suppress the T cell response. Furthermore, the cytotoxic effect of T cells was shown to be decreased by PD-L1-expressing neutrophils [28]. Similarly, peritumoral neutrophils are also reported to negatively regulate adaptive immunity through the PD-1/PD-L1 axis [29].

The clinical revolution of using CPI to treat cancer started with the approval of CTLA4 antibody, ipilimumab, by the United States Food and Drugs Administration (FDA) to treat metastatic melanoma patients [30,31]. Later, the monoclonal antibodies targeting the PD-1-PDL-1 pathway were also approved to treat a range of malignancies, such as lung, renal cell, liver and bladder cancers [32]. Particularly, ipilimumab—an antibody targeting the CTLA4 antigen, has been shown to significantly improve the survival outcome in patients with metastatic melanoma [31]. Ipilimumab treatment was observed to increase survival of melanoma patients by 20%, while the objective response rate in non-small cell lung cancer (NSCLC) was noted to be 19.4% after pembrolizumab administration [31,33]. Similarly, pembrolizumab and nivolumab, antibodies targeting PD1 are used as the first line therapy for non-small cell lung cancer (NSCLC). Akin to this, the CPI treatment has also shown to be beneficial for patients with other cancers types including head and neck squamous cell carcinoma (HNSCC), breast cancer and Hodgkin lymphoma [34,35]. Despite these promising outcomes of CPI therapy, not all cancer types respond equally to the treatment. For instance, although nivolumab showed promising outcomes in NSCLC patients in the phase I/II clinical trials, the phase III trial did not establish clinical efficacy of this treatment [36,37]. The overall response rate ranged from 5 to 30% for patients with triple negative breast cancer (TNBC) [38], while up to 40–45% of NSCLC patients responded to the treatment [33,39,40]. In addition, the metastatic form of castration-resistant prostate cancer and pancreatic ductal adenocarcinoma are mostly resistant to CPI therapy [41]. Furthermore, the outcome was also dependent upon the expression of PD-L1 by the tumor cells. One study in melanoma patients, showed that the 72% of patients who expressed PD-L1 responded to pembrolizumab treatment, while the response rate was lower (54%) in patients who did not express PD-L1 [40,42]. Similarly, two other studies on NSCLC patients demonstrated that the objective response rate to pembrolizumab treatment is higher (~45%) for PD-L1 positive patients [33,43]. These studies also indicate that pembrolizumab treatment has manageable side effects, and prior monitoring of the NSCLC patients for PD-L1 expression can be a judicious approach in deciding the use of the PD1/PD-L1 blocking antibody. On the other hand, Brahmer et al., reported that nivolumab treatment is devoid of cytotoxic effects and its efficacy was evident in squamous cell carcinoma patients with or without PD-L1 expression [44].

**Figure 1:**
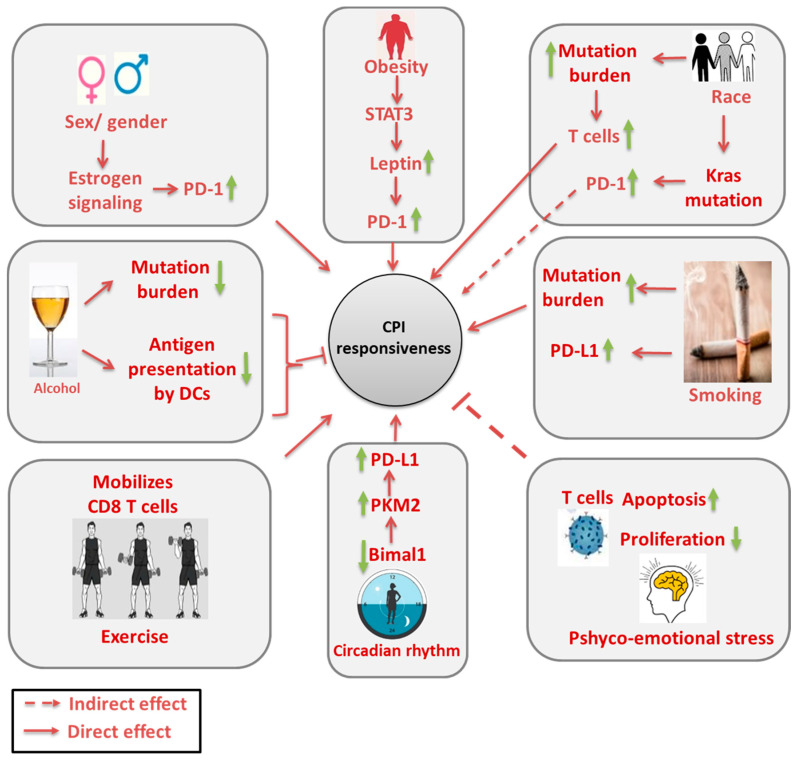
Various lifestyle and metabolic factors affect CPI effectiveness.

There is scope for treating patients with a combination of antibodies targeting multiple checkpoints. Concurrent combination of nivolumab targeting PD-1 and ipilimumab targeting CTLA-4 was reported to regress advanced melanoma by 80%. Other ongoing clinical trials using the combination of PD-1 or PDL-1 and CTLA-4 blockade have also shown significant improvements in outcome, highlighting combination treatment as a clinically efficacious approach [6,45,46,47]. The choice to use a particular mono- or combination therapy should be largely guided by examining the expression of biomarkers, such as PD-L1, that are indicative of better responses to CPI or by identifying factors that influence the efficacy of the CPI dose. Emerging evidence supports the contribution of cofounding factors both directly and indirectly affecting the immunotherapy outcomes. The aim of the present review is to provide an overall picture on the role of sociological factors, lifestyle habits and preexisting metabolic disorders (Figure 1) in modulating the CPI responsiveness. This in turn could help to understand the involvement of these stratifying factors on the interface of tumor–T cell interactions to harvest maximum therapeutic benefit in a clinical setting.

## 2. Roles of Sociological Factors and Sex/Gender in CPI Efficacy

Social differences can serve as disease and treatment modifiers [48]. Many drug interactions are previously acknowledged to be different in sociological variables [49]. In this section, we stratify the role of sex/gender, race and ageing on the outcomes of CPI.

### 2.1. Race

Race refers to genetic and phenotypic differences of humans. Accumulating evidence has shown that race differentially regulates the outcome of CPI therapy (Table 2). People of African origin are reported to have high risk of certain malignancies as compared to Asian Americans and Caucasians [50]. The high risk in the African population was primarily attributed to obesity-induced inflammation [51]. In addition to tumor incidence, race is known to impact the overall mutation rates in genes that play a pivotal role in CPI responsiveness. Particularly, the rates of EGFR and KRAS mutations are affected by the race of patients in NSCLC. EGFR mutations are more commonly found in Asian populations (32–57%) than other races, while African populations show a greater genetic diversity [52]. In a recent study by Sugiyama et al., the tumor microenvironment (TME) of EGFR mutated LUADs showed increased Treg infiltration, and a combination treatment of anti PD-1 with EGFR tyrosine kinase inhibitor erlotinib showed better anti-tumor effects [53]. On the other hand, Kras mutations in NSCLC are correlated with: increased PD-L1 expression in tumors, tumor mutational burden and TIL infiltration, resulting in superior response to anti-PD-L1/PD-1 treatment [54]. One study reported that African American (AA) patients with NSCLC respond better and display improved survival when treated with Nivolumab, although it was performed in a limited number of patients. High mutation burden in AA patients was attributed to the better responsiveness [55]. Another study by Nishino et al. compared the effect of nivolumab or pembrolizumab monotherapy in NSCLC patients from three racial-background - Asian, white and black [56]. They detected that 27 out of 143 white, zero out of six Asian, and one out of eight black patients responded to nivolumab or pembrolizumab and there was no significant difference in the pharmacokinetics of CPI therapeutic agents among the patients of different race. Similarly, an independent study found that the overall response rate was higher in Asiatic populations when compared with Caucasian patients [57,58]. Asiatic populations were shown to suffer from adverse effects of the CPI therapy, such as pneumonitis or pulmonary toxicity and hepatotoxicity [59]. On the basis of this evidence, surrogate mutations associated with racial differences may tailor the TME, concomitantly affecting the CPI efficacy. Therefore, it is essential to study CPI responsiveness in clinical trials specifically based on the race of patients to accurately determine its effectiveness (Figure 1).

### 2.2. Ageing

Cancer occurrence is relatively higher in older individuals. Reports suggest that >40% of the patients participating in the clinical trials are older than 65 years and this percentage is even higher for NSCLC patients (>60%) [67]. Older patients in the clinical trials are also underrepresented due to the toxicity associated with the treatment and most trials exclude patients with relatively poor responses [39,68,69,70,71,72]. Friedman et al. showed that patients over 80 years receiving CPI therapy showed significant side effects. Asymptomatic secretion of lipase was observed in 50% of patients administered with a combination of ipilimumab and nivolumab. Nausea, vomiting and diarrhea were most common symptoms in the nivolumab monotherapy group while a combination of ipilimumab and nivolumab prolonged survival, as compared with ipilimumab in this age group [63]. Similarly, Betof et al., have reported that 43% of the melanoma patients were identified with immune mediated toxicities, 9.8% of patients suffered colitis and 10% showed endocrine toxicity after the ipilimumab treatment [62]. The adverse effects were comparable in younger and older populations. Even though a few cases of older patients were noted with hypophysitis and thyroiditis, this did not reach the statistical significance. Overall survival in patients with >50 years of age in response to anti PD-1 immunotherapy was 22.9 months while it was 24.3 months for patients older than 75 years. Although there was a difference in overall survival among age groups, a multivariate cox regression model indicated these observations as statistically insignificant [62].

**Figure 2:**
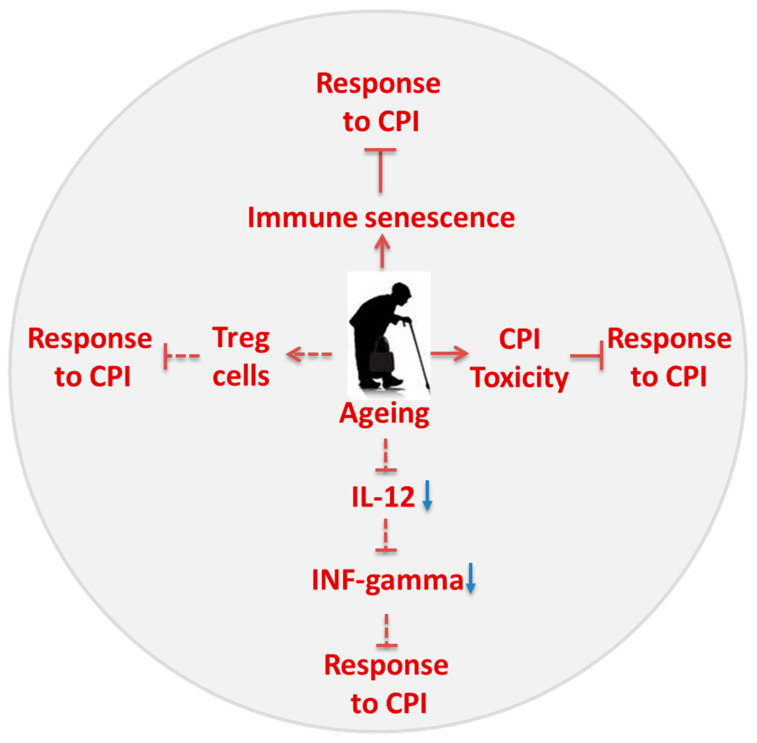
Ageing reduces responsiveness to CPI.

Many components of the immune system are misbalanced in older adults [73], and ageing is associated with decline in immune functions. T cells in older population show an impaired immunological signature, termed as immunosenescence, which contributes to a less efficacious response to immunotherapy. Immunosenescence is marked by decreased proliferation, effector functions, cytotoxicity of T cells and reduced capacity in responding to local antigen presenting cells by T cells, and increased accumulation of Treg cells, which inhibits the immune response and effector T cell activation [74]. Studies in older adults have shown that the CD8 T cell population declines partly due to loss of lymphopoietic stem cells [74,75]. Additionally, ageing is marked by upregulation of checkpoint marker proteins, such as Tim-3 and PD-1, and by a decrease in intracellular signaling through CD28 and IL-2 that are important for T cell activation [34]. Nishijima et al. reported that in the absence of functional T cell signaling, the CPI therapy finds itself with blunt ends in older adults. Patients with age >75 years did not demonstrate an improved survival rate when treated with the anti PD-1 antibody [76]. The failure of treatment is partly attributed to immunosenescence. Patients <75 years harnessed equal benefits as young adults. It appears that there is a need to decide the functional status of immune cells, such as CD8 T cells when treating the older patients with CPI [76]. At the functional level, T cells are marked by the upregulation of inhibitory receptors including PD-1 and Tim3, and also by the deficiency of the CD-28 co-stimulator and intracellular signaling necessary for T cell activation [77]. Ageing is also reported to diminish cytokine IL-2 production and its signaling, which plays a pivotal role in T cell activation [34]. Similarly, the secretion of another cytokine, IL-12, by dendritic cells was reduced in older adults which caused decreased INF-gamma secretion by T cells thereby impeding its cytotoxic activity [78]. Although the number of Naïve T cells was decreased in older cohorts, possibly due to reduction in the size of the thymus [79], the number of terminally differentiated CD8 T cell populations was found to be increased in lung cancer patients [80]. The frequency of Treg cells was also significantly increased in older people with HNSCC. As Treg cells induces an immunosuppressive microenvironment, tumor-associated immune suppression was shown to be less pronounced in these patients [81]. However, more studies are warranted in this line to precisely examine the metabolic changes in the tumor microenvironment—influenced by age—which in turn could influence the CPI efficacy (Figure 2).

### 2.3. Sex/Gender

Sex is a biological variable known to affect the innate and adaptive immune responses throughout the life of individuals. The immunological responses are variably guided by the age, reproductive status of individuals, secretion of sex hormones and gender [82]. Women, in general, respond more adequately to infections and vaccinations, as their innate immune system component is more robust than in males [82,83,84]. In the context of cancer, studies have shown that the mortality rates of men with melanoma, bladder and lung cancers are two-fold higher than women [85]. Indeed, sex hormones are known to regulate the expression of the PD-1 receptor on the surface of Treg cells. Polanczyk et al. investigated the influence of endogenous estrogen on PD-1 expression by using estrogen receptor knockout mice (ERKO). The PD-1 expression was mostly seen in the Tregs of wild type mice. However, the PD-1 level in Tregs of ERKO mice was significantly reduced, thus postulating that estrogen receptor signaling controls PD-1 expression. Accordingly, Treg functional activity was restored in PD-1 knockout mice when they were provided externally with estrogen. This study further showed that extracellular E2 enhanced estrogen receptor expression and promoted the expression of FOXP3, a prominent marker defining Treg cells [86,87]. The estrogen receptor (ER) is also known to be expressed by cytotoxic T cells but not by helper T cells [88]. On similar note, ER expression was observed to be cell-type dependent, i.e., ER-α is present on monocytes while ER-β is predominant in macrophages [89]. ER is also known to influence the development of dendritic cells (DC). ER-α and E2 are noted to be indispensable for the Irf4 dependent type I INF production by plasmacytoid DC (pDC), a distinct lineage of conventional DCs known to produce proinflammatory cytokines in abundance. pDCs from postmenopausal women exhibit a reduced TLR-7 response which was in part rescued by external ER supplement [90]. However, the effect of ER signaling in these immune cells and their role in CPI treatment response is yet to be studied. As hormone signaling is also known to affect other cancer types, such as prostate, endometrial, ovarian and colon cancer, it is imperative to determine the hormone response profile of individual tumor in the context of CPI therapy.

The existing literature draws conflicting conclusions on the selective benefit of sex in immunotherapy (Table 2). Fabio et al. summarized the published clinical data from 20 clinical trial consisting of 11,000 patients administered with anti-PD1 or anti-CTLA-4 therapy. They found that male patients derived a greater benefit of these therapies as compared to female patients. This study included 67% of males and 33% of females. The inclusion of more women in clinical trials was therefore suggested to extend the conclusions to female counterparts [91]. On the contrary, Wallis CJD et al. studied 23 randomized trials with 9322 men and 4399 female patients with advanced cancers of melanoma, NSCLC, head and neck squamous cell carcinoma (HNSC) and small cell lung carcinoma (SCLC) and concluded that both male and female patients derive equal benefit from the CPI therapy and there was no bias on the basis of sex [91,92]. On account of the existing dimorphism in immune responses on the basis of sexual differences, it is essential to conduct longitudinal mechanistic studies to outline the significant impact of sex hormone signaling on the functional profile of immune cells in TME and CPI efficacy (Figure 1).

Together, considering the fact that the difference in efficacy of the CPI response in young and aged populations is influenced by the functional status of T cells, presence of immune cells, such as Treg cells in the TME, and the toxicity profile, it is therefore necessary to carefully tailor the immunotherapy treatment for aged patients to obtain maximum therapeutic benefit.

## 3. Influence of Lifestyle on Outcome of CPI

Health is influenced by lifestyle habits [93]. In this section, we have summarized how lifestyle habits, such as smoking, alcohol consumption, diet and exercise modulate the human immune system directly or indirectly and thus have an effect on CPI efficacy (Table 3).

### 3.1. Smoking

Smoking is chronicled as the most prevalent habit among adults in the USA and is a robust risk factor for lung cancer development. Around 90% of lung cancer deaths are linked to previous smoking history [108]. Tobacco smoking is also a known risk factor for 16 other cancer types [109]. Tobacco smoking is conducive for tumor growth by inducing mutations in the tumor, modifying the tumor microenvironment and promoting pro or anti-inflammatory signaling [110,111,112]. Similar to obesity, several clinical findings in lung cancer have shown that patients with previous smoking history are more responsive to CPI therapy (Table 3). This improvement in outcome is mainly attributed to mutations in DNA, induced by carcinogens present in smoke, eliciting increased neoantigen burden in tumors leading to immunological recognition of tumor [113]. Furthermore, the faulty DNA damage repair pathway, which is frequently spotted in patients with smoking history, is associated with a higher mutational burden and neoantigen presentation in tumor cells [105,113]. Thus, the genomic landscape of a lung tumor is modified by tobacco smoking [36,105]. While the presence of neoantigens attracts T cells to the tumor, it also elevates PDL-1 expression in tumor cells, yielding improved responsiveness to CPI [36]. In addition to the increase in neoantigens, smoking alters the immune microenvironment of tumors in anatomic site dependent manner. In head and neck squamous cell carcinoma (HNSC), smoking creates an immunosuppressive microenvironment, as evidenced by the cytolytic score, an enriched interferon-γ (IFN-γ) signaling signature calculated from the RNA sequencing data [101,114]. In contrast, pro-inflammatory signaling is triggered by smoking in lung cancer [101]. One interesting study by Desrichard A. et al., indicated that the mutational burden and immune microenvironment of squamous cell carcinoma in smokers depends upon the anatomic site of the tumor, and proposed studying a few additional factors, such as PD-L1 expression and the T cell inflamed microenvironment, to treat patients with a smoking history [101]. These findings indicated that smoking impacts the mutational landscape and immune environment to alter the response to CPI treatment. Thus, smoking affects the CPI responsiveness by either changing the mutational burden or by altering the immunomodulatory balance of the TME [101].

The immune microenvironment of the tumor is widely classified into three subtypes: immune desert, inflamed and immune excluded types [115]. Immune desert tumors are marked by immunologic ignorance arising from either the absence of antigens or defects in antigen presentation by the loss of MHC I, B2 microglobulin or the loss of TAP-1 and TAP-2, which leads to death of functional T cells in the tumor stroma [116,117]. In such a non-inflamed tumor microenvironment, for example in melanoma and epithelial cancer with no functional CD8 T cells, tumors do not respond well to anti PD-1/PD-L1 immunotherapy [117]. In an immune inflamed tumor microenvironment, the tumor cells are in close proximity with the immune cells, such as monocytes, CD8/CD4 T cells and cells of myeloid origin and proinflammatory and effector cytokines [117,118]. Tumor types that exhibit an immune inflamed microenvironment include non-small cell lung, renal and rectal carcinoma. These tumors are infiltrated by antitumor T cells leading to increase in efficacy of anti PD-1/PD-L-1 therapy [118,119]. On the other hand, in immune excluded tumors, the immune cells do not reach the tumor as they are trapped in the stroma region. CPI administration can activate the CD8 T cells but the clinical response is uncommon as T cells are unable to infiltrate to the tumor [115,120]. It is to be noted, that smoking status differentially modulates the response of patients to CPI treatment. In a phase III randomized control trial with 1981 NSCLC patients, PD-1 inhibitors worked more efficaciously in patients with a smoking history while nonsmoking patients showed no survival benefit [121]. A similar meta-analysis in NSCLC, urothelial cancer and HNSCC showed that smokers benefited from anti PD-1/PD-L1 mono or combination therapy while nonsmokers benefitted from a combination of chemo- and immunotherapy [122]. One possible mechanism behind the increase in response to CPI treatment in smokers is related to an increase in PD-L1 expression by dendritic cells. Kerdidani D. et al., have shown that dendritic cells in the tumor environment of emphysema upregulated PD-L1 expression in smokers [123]. This increase in PD-L1 was mediated by oxidative stress created by smoking in the tumor microenvironment [123]. Other such evidence indicates that aryl hydrocarbon receptor (AhR) signaling, promoted by cigarette smoke, induced PD-L1 expression in lung epithelial cells [124]. Eighty one percent of patients with high AhR signaling responded well to anti PD-1 antibody pembrolizumab, while 75% patients with progressive disease exhibited low AhR in tumor tissues [124]. Similarly, immunomodulatory components of cigarette smoke can influence the tumor microenvironment and indirectly affect the CPI efficacy [101]. Nicotine is reported to induce MAP kinase channeled Cox2 expression which can lead to an inflammatory TME, thus indirectly affecting the functional status of immune cells and ultimately the efficacy of CPI [125]. On the other hand, crosstalk between smoking and HPV infection yielded a favorable outcome in HNSCC patients who received the anti PD-1/PD-L1 monotherapy. Smokers were found to be have HPV positive HNSCC, while tumors in nonsmokers were HPV negative [126]. Nicotine exposure is detected to decrease body weight by suppressing appetite, although the underlying mechanisms are not clear [127]. A study in mice has revealed that on exposure to cigarette smoke for 4 weeks, animals displayed reduced leptin levels, food intake, body fat and mass [128]. Leptin levels were previously observed to induce PD-1 expression and improve CPI efficacy [129]. Therefore, one can speculate that nicotine exposure in obese patients may yield better responsiveness. However, the pathophysiological mechanisms still remain unknown. Obviously, it is necessary to further dissect the effect of cigarette smoking on immune cells in the TME, leading to the alteration of CPI efficacy (Figure 1).

### 3.2. Alcohol Consumption

Approximately, 7% of patients with head and neck squamous cell carcinoma have a history of alcohol consumption and the adverse effect of alcohol is dependent on the age of the patient. In head and neck squamous cell carcinoma, the immune microenvironment of never smokers, never drinkers was found to be enriched with the PD-L1 and CD8 T cell infiltrate [96]. On the other hand, a low mutational burden was reported in head and neck cancer in patients with smoking and drinking history and the CPI treatment was functional in tumors with low PD-L1 expression [130,131]. Alcohol consumption is known to impact the innate and adaptive arms of the immune response. Primarily, alcohol causes a breach of the tight junctions in the gastrointestinal tract causing a leak in bacterial components, such as lipopolysaccharides into the blood stream [132]. Once in circulation, it activates endothelial cells setting off chronic inflammatory reactions [133,134]. Alcohol suppresses the recruitment of cells of the innate immune response, such as leukocytes [132], granulocytes [135], leading to aggravated bacterial infections. Alcohol abuse also causes a reduction in the phagocytosis activity of macrophages by altering the surface receptors required for adherence and production of reactive oxygen species necessary for pathogen killing [136]. Thus, consumption of alcohol is connected to the intricate functions multiple immune cells and molecules involved in CPI therapy (Table 3).

Alcohol also impairs dendritic cell number by interfering with their differentiation and ability to stimulate T cells [137]. In addition, alcohol suppresses the ability of antigen presentation of dendritic cells by reducing the expression of CD80 and CD86 cell surface receptors that are essential for antigen presentation to T cells [138]. Alcohol also decreases stimulation of naïve CD4 T cells to INF gamma producing Th1 T cells by reducing IL-12 production by dendritic cells [134]. Chronic alcohol intake also enhances the production of TNF alpha, a well-studied proinflammatory cytokine [139], while acute alcohol exposure is found to promote opposite effects, i.e., suppresses the cytokine and chemokine response [140]. CD8 T cells in patients with alcoholic hepatitis were found to have reduced cytotoxic functions and reduced activation [141]. Mechanistically, CD8 T cells in patients with alcoholic liver cirrhosis were found to have decreased function due to the absence of CD28 co-stimulatory molecule and decreased responsiveness to exogenous IL-2 due to impairment of IL-2 binding receptor [142]. Although there is a scarcity of reports showing the direct impact of alcohol consumption on CPI efficacy, T cells in patients with acute alcoholic hepatitis were shown to have higher expression of PD-1, PD-L1, Tim-3 and galaction-9 levels as compared with T cells from non-alcoholic individuals [143]. This may help in a way to extrapolate the possible effects of alcohol consumption in the efficacy of the CPI response. In light of the existing evidence, more studies are needed to establish the impact of alcohol consumption on the expression of immune checkpoint proteins such as PD-L1, which to a large extent dictate the CPI efficacy (Figure 1).

### 3.3. Diet

Appropriate macronutrients, micronutrients, fibers and energy sources are essential to build a healthy immune system [144]. Poor or inadequate nutrition often results in compromised immune responses which results in a predisposition to infections [145]. Consumption of fruit juices and hyper caloric breakfast is reported to result in a decrease in inflammatory cytokine IL-17 [146]. Further, IL-10 levels were found to be increased in a group of children who followed the Mediterranean diet while IL-17 was increased in saliva of children who had junk food. IL-10 cytokine is predominantly produced by FOX P and Tregs [147,148]. Baseline levels of IL-17 are reported to predict toxicity in melanoma patients treated with ipilimumab and develop colitis/diarrhea during the course of treatment. The same study also reported that high baseline IL-10 levels were correlated with tumor relapse [149]. Yamazaki N et al. noted that in melanoma patients treated with anti PD-1 (nivolumab), serum IL-10 levels were higher in patients with an objective response rate [150]. TIGIT has been shown to inhibit the T cell responses by inhibiting dendritic cell maturation and inducing the release of immunosuppressive cytokine IL-10 [14,15]. IL-10 has been shown to restrict T cell proliferation by reducing the INF gamma and IL-2 synthesis, thereby inhibiting proliferative and cytokine responses in T cells [151]. IL-17 is reported to be secreted by the LAG-3 positive T cells [152] and IL-17 mRNA expression is positively correlated with LAG-3 T cells in gastric cancer [100]. Thus, patients with IL-17 overexpression may benefited more from the treatment of LAG-3 inhibitors. One study showed that dietary methionine restriction plays an important role in reprogramming the tumor-associated macrophage towards the M1 functional phenotype, through the mTOR pathway, making them more tumoricidal. It leads to enhancing the effect of the anti PD-1 immunotherapy response in prostate cancer [98]. At present, there is a scarcity of literature linking the role of dietary intake with CPI response. However, certain dietary components such as flavonols [153], stilbenes [154] are reported to reduce inflammation by suppressing the NFkB activity. Isoflavones as genistein is known to function as an antioxidant and suppress the NFKB activity when supplemented with arachidonic acid in the diet [155]. Inflammation is regarded as an important player in modulating the TME and ultimately affecting the CPI efficacy (Table 3), therefore, it would be worth investigating to extrapolate the presence of inflammatory component to the efficacy of CPI therapy.

**Figure 3:**
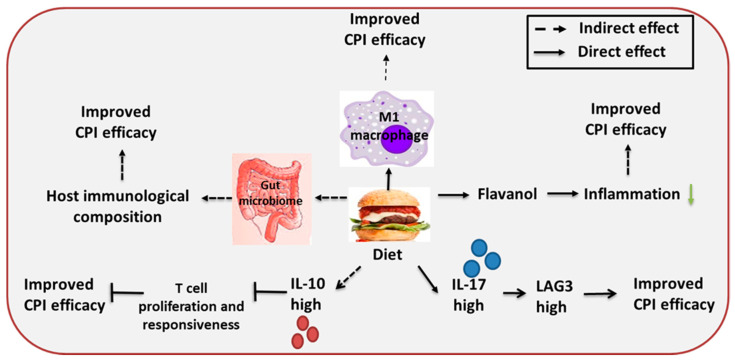
Effect of diet on CPI response.

The human gut microbiome is composed of various microorganisms and reported to play an important role in human health and disease [156]. Gut microbes are reported to affect the immune cell profile of the local mucosal and peripheral immune system [157]. Germ free (GF) mice are found to have smaller mesenteric lymph nodes (MLN), decreased number of plasma cells, CD4 T cells and lowered IgG production, which collectively impairs the intestinal barrier function [158]. In addition, commensal bacteria are known to impact CD4 T cells and the maintenance of the Treg/Th17 balance. GF mice are also characterized with increased Treg cells [159]. Additionally, bacterial metabolites are reported to stimulate dendritic cell phagocytosis in bone marrow [160]. Multiple studies document that the gut microbiome profoundly impacts the immunotherapy response in a range of malignancies, such as melanoma, hematologic cancer NSCLC, renal cell carcinoma [161]. Experimental data in a mouse sarcoma model suggest that B. fragilis (Bf) enhances the anti-CTLA-4 efficacy by promoting cross reactivity of T cells between bacterial and tumor antigens [99]. Fecal microbial transfer from the human melanoma responder undergoing anti-CTLA treatment to germ free mouse enriched Bf and its abundance was negatively correlated with tumor size in response to anti-CTLA treatment [99]. In melanoma, Bifidobacterium supplement was reported to enhance tumor infiltration and INF-γ production by CD8 T cells [162]. It was further noted to enhance anti-PD1 efficacy by activating intratumoral DC cells [162]. Studies in NSCLC and renal cell carcinoma show that the bacterial species, Akkermansia muciniphila, was enriched in stool samples of patients receiving anti PD-1 immunotherapy treatment [163]. Oral supplementation of Akkermansia muciniphila was noted to restore the anti PD-1 efficacy by enhancing IL-2 secretion and CCR9^+^CXCR3^+^CD4^+^ T lymphocytes in mouse tumors [163]. Apart from the role in enhancing CPI therapy by influencing the activity of immune cells, gut bacteria have also been shown to mitigate the immunotherapy treatment associated toxicity. For instance, the presence of bacteria from the Bacteroidetes phylum in stool samples of melanoma patients receiving anti-CTLA4 CPI, were less prone to treatment induced colitis [164]. Further, the impact of bacteria on the efficacy of the CPI regime is complicated by the mechanistic elements associated with bacterial species diversity and interaction with host and functional attributes should be weighed with caution. For instance, there could be variation at the strain level in bacteria of the same species, thus yielding divergence in the immunological impact on host [165]. In addition, there is a need to study the variation in the composition of bacterial diversity over the course of treatment and pathologic state of cancer progression to draw a meaningful inference for personalized cancer therapy (Figure 3).

### 3.4. Exercise

Exercise is the supportive care provided for the cancer patients to maintain their wellbeing. Such adjuvant approaches are noted to reduce the side effects of CPI treatment and help in fatigue management [35,166,167]. Exercise is conducive to maintain good health in cancer patients by enhancing the self-efficacy and wellbeing [168]. Simpson et al. have reported that exercise promotes expansion of tumor targeting T and NK cells in healthy donors and the T cells isolated from exercise-primed individuals were better suited for ex vivo expansion [94] (Figure 1). Additionally, exercise was induced to enrich the hematopoietic stem cells (HSC) in the blood [94]. This evidence shows the impact of exercise on the expansion and activity of immune cells, which may have a crucial impact on CPI effectiveness. However, more direct mechanistic evidences are required to justify the role of exercise in enhancing the CPI responsiveness (Table 3).

### 3.5. Circadian Rhythms

The circadian clock or rhythm is an internal timing system used by organisms [169]. In mammals, the superchiasmatic nucleus (SCN) in the hypothalamus works as a pacemaker regulating the peripheral clocks in cells [170,171]. It is well established that intrinsic timings dictated by circadian rhythms regulate the functional aspects of the innate and adaptive immune system cells [172]. This temporal gating modulates a range of immunological functions such as autoimmune diseases induced by T cells and lymphocyte trafficking in lymph nodes [173]. Wenjun Deng et al., has demonstrated that circadian rhythms control the immune checkpoint pathway in septic shock induced by infections. They also showed that deficiency of Bimal1, which is a core circadian gene, increased lactate production and PKM2 expression which was required to upregulate PD-L1 in a STAT-1 dependent manner. The increased PD-L1 suppressed apoptosis in T cells promoting exhaustion and immunosuppression [106]. Chloé C. Nobis et al., have shown that the circadian clock affected the functional response of CD8 T cells to antigen presentation by dendritic cells, which is a core aspect of the immune response against pathogens and cancer cells. This early T cell response was shown to impact T cell receptor signaling such as activation, proliferation and effector functions [174]. Thus, the influence of circadian rhythms on the functional aspects of T cell development, response to antigens, and trafficking effector functions is well appreciated in the existing literature [174,175,176]. Although there is no direct evidence eloquently describing the mechanistic interplay between circadian rhythms and the immunotherapy response, based on the existing reports featuring its involvement in modulating the innate and adaptive arms of immune system, it would be worth deciphering the link in the clinical setting.

### 3.6. Psycho-Emotional Disturbances

Although there is no direct evidence reporting the impact of immunotherapy on mood, anxiety and cognitive impairment [177], studies have indicated the role of innate and adaptive immune dysfunction in the development of depression [178]. Peripheral blood of patients with major depression was shown to have a significant increase in inflammatory cytokines, such as tumor necrosis factor (TNF)-alpha, interleukin (IL)-1, IL-6 and other acute phase proteins related to inflammation [107,179]. In addition, depression and stress were shown to decrease proliferation, and promote apoptosis in T cells. Treg stimulation, by low dose IL-2, is postulated to help a subset of patients with depressive disorders [107]. Thus, so far there is no convincing direct evidence on the impact of depression and stress on immunotherapy responsiveness. Further study is warranted to consolidate the involvement of psychological stress on the outcomes of the immunotherapy response.

## 4. Effect of Metabolic Disorders on CPI Treatment

Immune reactions and cellular metabolic changes are closely linked. Alternation in cellular metabolism is evidenced to impact the magnitude of immune responses [180]. In this section, we have evaluated the direct impact of metabolic diseases such as obesity on the efficacy of the CPI response. We also discuss the consequences of the CPI response in terms of metabolic changes (Table 4).

### 4.1. Obesity

Obesity is one of the most prominent health issues in the United States, and it is globally ranked as the sixth risk factor for disease predisposition [192]. Body mass index (BMI) is the routinely used to measure the burden of obesity. According to WHO specification, a BMI ≥ 30 is defined as being obese [193] and can lead to other comorbidities such as hypertension, heart disease and cancer [193,194,195]. Obese patients remain in a chronic inflammatory state as adipose tissues secrete high levels of adipokines as leptins, TNF-α, IL-6, and IL-8 [196]. This inflammatory state is known to change the immune landscape by several mechanisms, including a decrease in thymic function, polarization of macrophage to M2 macrophage and prolonged stimulation from toll like receptors (TLRs) expressed by antigen presenting cells. Inevitably, obesity also modulates the tumor response to CPI treatment [197].

Several lines of evidence have reported obesity to play a profound role in the response to CPI treatment. Studies in metastatic melanoma and renal cell carcinoma have shown obese patients survive significantly longer when treated with CPI [181,198,199]. Similarly, Naik G.S. claimed that obese patients are at lower risk of disease progression or mortality when treated with anti PD-1 monotherapy or a combination therapy of anti PD-1 and anti-CTLA-4 [183]. Because BMI does not efficiently indicate the lean mass or adiposity, this study used serum creatinine as a surrogate marker of muscle mass to study the response to CPI treatment. Interestingly, they found that the outcome of CPI treatment was evident only in males, while females with lower creatinine levels did not benefit from CPI treatment, suggesting a gender based discrepancy to CPI treatment [183]. In contrast, Xu et al. performed a retrospective meta-analysis of approximately 4200 patients and found that obesity is associated with better outcome to CPI in both males and females [200]. Another study of melanoma patients administered with a combination of pembrolizumab and nivolumab, showed lowered distribution of CPI antibodies in the body circulation of obese individuals resulting in overexposure and excessive toxicity without any difference in tumor response [201]. The overall, objective response rate was 50% among the patients with toxicity, which is in agreement with the existing literature [10]. However, the mechanistic underpinnings associated with this differential response were not demonstrated. One study has shown that obesity leads to tumor progression by increasing T cell ageing, a phenomenon in which the cytotoxic function of T-cell is compromised [202]. The T cell dysfunction in obese patients was driven by PD-1 overexpression [105]. As STAT3 is known to promote PD-1 expression in T-cells, the increased level of leptin in obese patients elevated PD-1 expression via STAT3 activation. Lack of leptin signaling was observed to rescue the T cells from exhaustion in diet induced obesity animal model [203]. Furthermore, this study also showed that high BMI was associated with a better survival outcome in lung, melanoma and ovarian cancer patients [129]. Moreover, it has been addressed that pro-inflammatory cytokines increase expression of neoepitopes and activate the T cell response to tumors [204], suggesting that the CPI response of tumors in obese patients may involve multiple other factors.

CPI responsiveness for patients with an obesity condition was further influenced by the gender. Male patients benefited more than females in metastatic melanoma [181]. Estradiol concentration was found to be higher in obese males than in women due to increased aromatase activity of the adipose tissue. It was shown to positively influence the survival of patients [205]. Race is also discerned to impact the BMI score [206]. With the same level of body fat, African Americans tend to have lower BMIs than Caucasians, while the BMI of Chinese, Indonesians, and Ethiopians was lower than Caucasians [207]. Overall, although the obesity paradox, in the context of CPI response is well established, it is imperative to obtain deeper mechanistic insights and study the tumor microenvironment under the CPI treatment in obesity patients (Figure 1).

### 4.2. Diabetes

An intricate relationship exists between diabetes and cancer as patients with type 2 diabetes are at higher risk of developing multiple malignancies including breast, endometrial, bladder, pancreatic and colon cancer [208,209]. On the other hand, patients under insulin or insulin analogues treatment have increased risk of colorectal [210] and skin [211] cancer development. A study by Eikawa et al., used syngeneic melanoma mice model to show that metformin, drug used for lowering the blood glucose level in diabetic patients, increases T cell infiltration into tumors and exerts antitumor immunity by directly enhancing T cell functionality and protecting them from exhaustion (154). This study also found an improved antitumor effect when vaccine or cellular therapy was combined with metformin treatment [212], raising the possibility that diabetic patients under a metformin regimen may benefit from CPI therapy. Additionally, diabetes is associated with a higher BMI of the patients, and BMI positively correlates with IFN-γ production by CD8 T cells [129]. Therefore, it can be speculated that high BMI diabetic patients may have better outcomes under CPI treatment.

**Figure 4:**
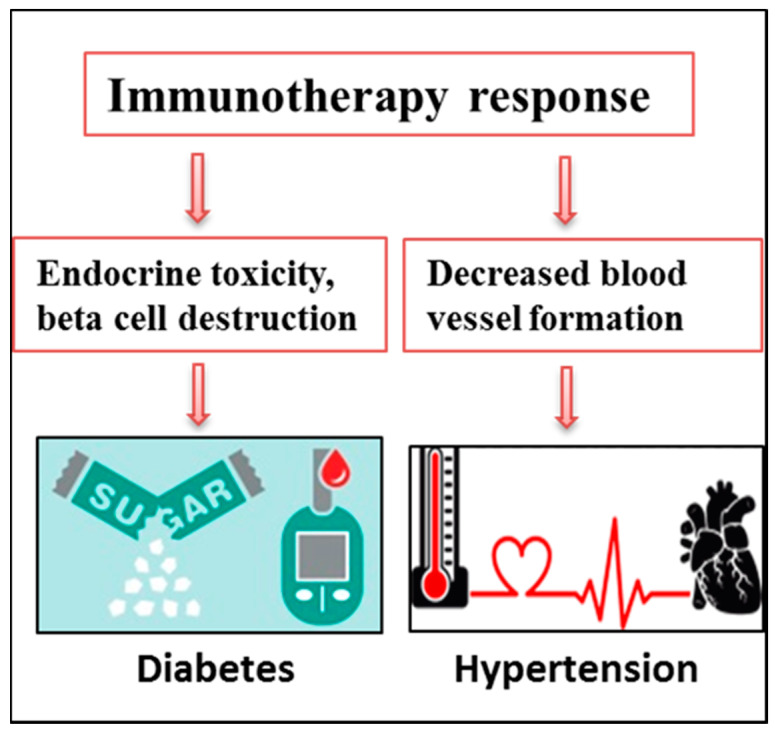
Secondary effects of immunotherapy response.

As the CPI treatment (CTLA-4, PD-1 and PD-L1 inhibitor) unleashes the T cell inhibition to destroy the tumor cells, it is also reported to cause endocrine toxicities including, diabetes, hypothyroidism or hyperthyroidism [44]. Diabetes onset was prominently noted after the initiation of the CPI treatment. A study showed that patients who had normal blood glucose levels before treatment showed insulin deficiency leading to diabetic ketoacidosis and progressing to type I diabetes after CPI administration [213]. In addition, the median age of patients diagnosed with type I diabetes under the CPI treatment was much higher than estimated, making the involvement of CPI more possible [214,215]. It was also observed that knowing the family history of diabetes or autoimmunity helps to a great extent in predicting the predisposition of the patients to CPI induced diabetes [186]. On a similar note, a few other studies have documented the similar incidences of rapid diabetes progression [216]. HLA genes were previously shown to be responsible for type I diabetes [217]. Interestingly, Matsumura et al., demonstrated the importance of identifying the HLA genotype before considering treatment of diabetic patients [218]. It is also imperative to check the status of autoreactive T cell by clonotype analysis for excluding patients who are at high risk of developing auto inflammatory toxicities from CPI treatment [218,219]. A close follow-up for the blood glucose history before and during the treatment is important to predict the onset of diabetes as its progression is much faster while under the CPI regime. Together, the awareness of physicians and effective management is necessary to mitigate the unwanted secondary responses (Figure 4).

### 4.3. Hypertension

Hypertension is specified as another secondary consequence of CPI therapy. Particularly, angiogenesis inhibition treatment by bevacizumab, was found to promote hypertension in colorectal cancer [220,221]. Nitiric oxide mediated contraction of blood vessels leads to excretion of sodium ions, ultimately causing hypertension [222]. A study has noted that Pembrolizumab, when given with Linvatinib, often causes hypertension in endometrial cancer [188]. It was further marked that hypertension was mostly aggravated in grade three endometrial cancer patients as a secondary consequence [188]. The mechanistic details of underlying signaling and the possible predictive markers are not well documented in the existing literature. It is therefore necessary to complete a better risk assessment for the possible prediction of secondary abnormalities such as hypertension before the actual start of CPI (Figure 4)**.**

## 5. Conclusions and Future Directions

Checkpoint blockade therapy (CPI) has impacted on extending the quality of life for cancer patients. It primarily acts to unleash the barriers of the immune system to recognize and destroy the cancer cells. Presently, the use of CPI is being accelerated in a wide range of cancers. However, its overall effectiveness remains uneven. The deviations in the overall efficacy of CPI seems to be determined by an array of factors such as mutational burden, anatomic site of tumor, composition of the immediate microenvironment, pathways modulating the expression of the CPI ligand–receptor pair, infiltration of cytotoxic T cells and other immune cells which augment or influence the killing process. To achieve the optimum efficacy of CPI, it is crucial to review the factors directly or indirectly impacting its effectiveness to boost its inclusive adequacy. As a step towards this goal, it is essential to consider into practice the impact of environmental factors or the inherent metabolic diseases pervading in the patients before prescribing the drug. In line with this idea, we also note secondary effects of CPI most commonly seen among patients. The existing literature documents incidence of diabetes and hypertension in the form of endocrine aberrations in response to CPI. The occurrence of these side effects needs to be closely monitored to enhance the quality of life among patients. Further, the physiology of patients, the inherent metabolic errors such as obesity and cultivated habits including smoking, exercise and alcohol consumption impact the CPI effectiveness. In particular, we observe factors with duel involvement in affecting the CPI response such as diabetes, smoking and ageing. The context dependent role of these factors may be attributed to the biology of the individual patients, the nature and anatomic location of the tumor and its unique cellular and metabolic fingerprint. For metabolic diseases like diabetes, and obesity it remains pivotal to identify the biomarkers for better responsiveness. At the same time, it is necessary to know the role of these variables in modulating the TME, which ultimately affects the CPI efficacy. Similarly, markers for better tolerance of CPI treatment needs to be identified for aged cohort. Furthermore, the individual state of immune system is influenced by lifestyle habits such as smoking and alcohol consumption that has a role in guiding the CPI responsiveness. We need more trials in establishing the role of these external agents to have better conclusive evidence. Additionally, to minimize the inherent toxicity and unwarranted secondary effects, it is needed to have longitudinal profiling of biomarkers for a better measure. Together, better understanding of the factors responsible for proper conditioning of the individual immune system is of paramount importance to harvest the maximum benefit and at the same time minimize the deleterious effects of the CPI.

## Figures and Tables

**Table 1:** Key genes involved in checkpoint blockade immunotherapy (CPI) responsiveness.

Gene	Expression Profile	Modulators	Role in CPI Responsiveness	Predictive vs. Prognostic Value	Reference
**PD-1**	TIL	PTEN, PI3K-Akt pathway, STAT3	Inhibits T cell proliferation	Predictive and prognostic	[6,7]
**PD-L1**	Tumor cells Macrophage, stroma cel	MAPK and PI3K or Akt pathways	On interaction with PD-1 inhibits T cell proliferation	Predictive and prognostic	[8,9]
**TIM3**	CD4, CD8 memory T cells, DC, NK cells, monocytes	IL-2, TNF-α, IFN-γ	Promotes T cell dysfunction/exhaustion, Tim3^+^ Tregs correlated with metastasis disease	Prognostic	[10,11]
**LAG-3**	CD4, CD8 T cells, NK cells, Treg cells	IL-10 and TGF-β1	Promotes Treg mediated suppression Inhibits effector T cell proliferation	Prognostic	[12,13]
**TIGIT**	Tregs, TILs (CD8), DCs	IFN-γ and IL-17	Suppresses anti-tumor immunity by dampening CD8 T cell function via Tregs	Prognostic	[14,15]
**CTLA**	Tregs, CD4 T cells	IL-2, PI3K pathway, Bcl-XL	Mediates immunosuppressive signaling by blocking co stimulatory CD28 receptor, inhibits T cell activation	Predictive and prognostic	[16,17]

**Table 2:** Effect of sociological factors in modulating CPI response.

Factor Influencing	Effect on Check Point Inhibitor Treatment	Mechanism Involved	Cancer Type	HR(CI), *p*-Value of CPI Response *	Reference
Sex/gender	No correlation	--	Advanced Squamous-Cell Non-Small-Cell Lung Cancer	--	[43]
	For anti CTLA treatment PFS longer in males	Men have high CD8 T cells expression	NSCLC	HR Male: 0.77 (95% CI 0.63–0.94), *p* = 0.012 HR Female:0.89 (95% CI 0.76–1.05) *p* = 0.16	[60]
	Longer survival in females for anti CTLA treatment	--	Melanoma	HR Female: 0.80 (95% CI 0.68–0.94) *p* = 0.006	[60]
	No correlation	--	Advanced gastric and gastroesophageal junction adenocarcinoma	--	[61]
Race	AA have high response rate to Nivolumab	AA have higher mutational burden	Lung cancer	--	[55]
Ageing	Adverse effects in elders in melanoma	Not clear	Melanoma, Metastatic melanoma	--	[62,63]
	No correlation	--	NSCLC	--	[64]
	Elders have less toxicity of CPI therapy	Not clear	Melanoma, non-small cell lung cancer, and renal cell carcinoma	--	[65,66]

* Hazard Ratio (HR) calculated by comparing CPI treated and untreated group. AA: African American; NSCLC: non-small cell lung cancer

**Table 3:** Influence of lifestyle changes on CPI response.

Factor Influencing	Effect on Check Point Inhibitor Treatment	Mechanism Involved	Cancer Type	HR(CI), *p*-Value of CPI Response *	Reference
Exercise	Improved response to immunotherapy	Exercise lowers the expression of PD-1 on T cells mobilizes more CD8 t cells	Blood cancer	--	[94]
	Low symptom burden	Not clear	Metastatic melanoma	--	[95]
Alcohol Consumption	Improved response to immunotherapy	High intratumoral T cell infiltrate, overexpression of PD-L1 in never drinkers	Oral squamous cell carcinoma	--	[96]
	No significant correlation between alcohol consumption and PD-L1 expression	--	Oral squamous cell carcinoma	HR 1.2 (95% CI 0.91–1.71) *p* = 0.15	[97]
Diet	Enhanced immunotherapy effect	Enhanced anti-tumor capacity of TAM	Prostate and renal cell carcinoma	--	[98]
		Gut microbiome modifies host immunity	Melanoma	--	[99]
		High LAG-3 induced by IL-17	Gastric cancer	--	[100]
Smoking	Controversial reports in HNSC and LUSC	Increased mutation rate. Decreased immune cell infiltration and poorer survival in HNSC, reverse in LUSC	head and neck (HNSC) and lung (LUSC) squamous cell carcinoma	LUSC: HR 1.02 (95% CI 0.71–1.46) *p* = 0.92	[101]
	High responsiveness	High mutation rate	Lung adenocarcinoma, NSCLC	NSCLC: HR 0.86(*p* = 0.61)	[58]
				NSCLC: HR 0.81 (95% CI 0.27–2.43) *p* = 0.71	[102]
				NSCLC: HR 0.45 (95% CI 0.22–0.92) *p* = 0.02	[103]
				NSCLC: HR 0.71 (95% CI 0.63–0.82) *p* < 0.00001	[104]
				NSCLC: HR 0.15 (95% CI: 0.06–0.39) *p* = 0.0001	[105]
Circadian rhythms	No direct evidence	Decreased Bimal-1 causes high PD-L1 expression	Not reported in cancer condition	--	[106]
Psyco-emotional changes	No direct evidence	Depression and stress decreases proliferation, increases apoptosis in T cells	Not reported in cancer condition	--	[107]

* Hazard ratio (HR) calculated by comparing the presence and absence of influencing factor.

**Table 4:** Effect of metabolic diseases on CPI response.

Factor Influencing	Effect on Check Point Inhibitor Treatment	Mechanism Involved	Cancer Type	Reference
Obesity	Improved survival	Not clear	Melanoma	[181]
	High response rate	Leptin in obesity cases promoted PD-1 expression on T cells	Melanoma, colorectal cancer	[13,129]
	Acute limiting toxicity (ALT)	Low distribution of drug, high exposure	Melanoma	[182]
	Worst Survival	Lower creatinine levels in obese females	Melanoma	[183]
Diabetes	Diabetes is secondary to immunotherapy	Endocrine toxicity, beta cell destruction	NSCLC Melanoma	[184,185,186,187]
Hypertension	Secondary to immunotherapy	Inhibition of blood vessel formation	Colorectal cancer, Melanoma, Endometrial cancer	[188,189,190,191]
